# Beyond Ipilimumab: a review of immunotherapeutic approaches in clinical trials in melanoma

**DOI:** 10.1093/immadv/ltaa010

**Published:** 2020-12-18

**Authors:** Gary Middleton

**Affiliations:** Institute of Immunology and Immunotherapy, University of Birmingham, Birmingham, UK

**Keywords:** melanoma, neoadjuvant, cytokine, vaccine, neoantigen

## Abstract

In this first in a series of ‘Trials Watch’ articles, we briefly review a highly selected set of clinical trials that are currently recruiting or about to open to recruitment in melanoma, the disease first transformed by the introduction of immune checkpoint blockade inhibitors (ICI). We place equal emphasis on phase I/II studies investigating the activity of biologically compelling novel immunotherapeutics, and on randomised trials of ICI with and without novel agents, as these latter studies optimise the standard-of-care use of ICI, and determine whether novel agents become part of the approved therapeutic armamentarium. We do not consider here combination therapy with other checkpoint antagonists or agonists besides combination of anti-PD-1/PD-L1 monoclonal antibodies (mAbs) with anti-CTLA4 mAbs, as these will be reviewed in a subsequent article in this series. A glossary of agents to be discussed is found at the end of this article.

## Introduction

Following clinical trial results in 2011 that demonstrated its remarkable potential to induce durable complete responses in patients with metastatic melanoma, Ipilimumab (anti-CTLA4 mAb) was approved for standard-of-care adjuvant treatment of resected stage III melanoma in 2015. Two years later, based on the results of the CHECKMATE 238 trial, Nivolumab (anti-PD-1 mAb) was approved for the adjuvant treatment of patients with melanoma with involvement of lymph nodes or in patients with metastatic disease who have undergone complete resection. Jeffrey Weber updated these results at ESMO 2020 [1] and reported a relapse-free survival (RFS) hazard ratio for disease recurrence of 0.71 with median RFS 24.1 months for Ipilimumab, and 52.4 months with Nivolumab. At 48 months, there was an absolute difference in RFS of 11 percentage points. There was however no difference in 4-year overall survival (OS) at 77–78%. Liu and colleagues compared neoadjuvant with adjuvant T_reg_ depletion or anti-PD-1 mAb/anti-CD137 mAb in two breast cancer mouse models [2]. There were significantly greater numbers of mice with long-term survival when treated with neoadjuvant ICI (NACI). This differential survival was specific to immunotherapy as it was not observed with paclitaxel. Moreover, it was associated with strong increases in all organs examined of tumour-specific proliferating CD8+ T cells with enhanced effector function. One likely advantage of neoadjuvant immunotherapy is enhancement of systemic T-cell responses to tumour antigens when detectable tumour is still present. NACI also has the added benefit of potentially sparing patients total lymph node dissection in those with ‘major pathologic response’ (MPR) in the index lymph node as reported in the PRADO trial at ASCO 2020 [3], reducing the need for adjuvant therapy in those with MPR (MPR defined as a maximum 10% viable tumour cells remaining in their index lymph node).

## Reducing the risk of post-surgical relapse of resected late-stage (III/IV) melanoma using combination checkpoint blockade

Although the use of adjuvant anti-PD-1 mAb ICI improves outcome in resected stage III/IV melanoma, relapse remains a major problem. There is considerable scope for improvement, particularly to improve long-term overall survival, possibly through the use of NACI, and furthermore to potentially exploit the response to NACI to finesse the treatment options post-operatively. Thus, CheckMate 7UA (NCT04495010) is a pivotal three-arm trial which will randomise 657 patients and explore different treatment options for stage III melanoma. Specifically, it will compare the current standard of care (adjuvant Nivolumab after tumour resection) with an alternative regimen involving a neoadjuvant Nivolumab plus Ipilimumab combination, followed by adjuvant Nivolumab. Furthermore, a third arm will feature neoadjuvant Nivolumab plus Ipilimumab, combined with post-operative pathologic response-directed observation, supplemented with adjuvant Nivolumab for patients without MPR. RFS is the primary end point and the neoadjuvant regimen uses low-dose Ipilimumab in two 3-weekly cycles pre-operatively, a regimen found to have the optimal balance of favourable tolerability with high levels of pathological response [pathologic complete response, defined as no tumour cells remaining, was 57%] in the OpACIN-neo trial [4]. CHECKMATE 7UA is due to open to recruitment early 2021. A SWOG (formerly the Southwest Oncology Group) study (S1801 – NCT 03698019) on pembrolizumab (anti-PD-1 mAb) randomises stage III/IV patients with resectable disease to one of two regimens, involving either three 3-weekly cycles of neoadjuvant ICI followed after tumour resection by 15 cycles of adjuvant therapy, or alternatively to 18 cycles of adjuvant ICI treatment.

## Controlling post-surgical relapse of stage II disease with checkpoint blockade

Although disease relapse is less common in stage II than in stage III disease, the higher relative frequency of stage II disease makes relapse after surgery a significant healthcare burden. Currently, however, patients with stage II disease do not receive adjuvant therapy following surgical resection. Two double blind placebo-controlled trials are investigating the impact of adjuvant Nivolumab (CHECKMATE 76K) or pembrolizumab (anti-PD-1) (KEYNOTE-716) after resection of stage IIB/C melanoma. If these trials are positive, potentially all patients undergoing resection of IIB/C disease could receive adjuvant therapy. However, since many such patients would be destined never to relapse, they would risk receiving unnecessary therapy. An elegant solution to this problem may be provided by the DETECTION study, where the focus is on early treatment of minimal residual disease (MRD) rather than blanket adjuvant therapy. The approach exploits a blood biopsy to detect circulating tumour DNA (ctDNA), which can be a simple way to test for MRD, based on assessment of BRAF, NRAS, or TERT mutations that are present in more than 75% of melanoma patients’ tumours. ctDNA for these mutations in IIB/C resected, mutation positive patients will be assayed every 3 months for the first 3 years and then 6 monthly in years 4 and 5. Those patients in whom ctDNA becomes positive, indicative of early molecular relapse, will be centrally randomised (blind to investigator and patient) to either continued clinical surveillance with investigator choice therapy at clinical or radiological relapse, or Nivolumab 4 weekly for 2 years. This trial therefore aims to bring a blood biopsy molecular stratifier into clinical decision making around post-operative therapy choices and is an important study in exploring whether ICI treatment in the MRD setting can significantly deflect the natural history of disease. ‘First patient first visit’ is planned for early next year.

## Stimulatory cytokines

A number of approaches are being pursued to therapeutically exploit immunostimulatoroy cytokines in melanoma, particularly involving IL-2 and IL-15. IL-2-focussed studies build on the previous observation that high-dose IL-2 has single agent activity in melanoma but is associated with significant toxicity. One such approach focuses on Bempegaldesleukin, a modified IL-2 which acts as a CD122-preferential IL-2 agonist that signals via the dimeric IL-2βγ receptor expressed by T effector and natural killer (NK) cells. This avoids activation of T_reg_ cells, which occurs via IL-2αβγ receptor signalling. Preferential signalling is achieved via conjugation to six polyethylene glycol chains, with slow release of the polyethylene glycol chains increasing drug half-life, and resulting in sustained signalling, allowing less frequent dosing and improved tolerability compared with high dose IL-2. Bempegaldesleukin is therefore designed to selectively avoid T_reg_ stimulation and instead stimulate T effector cells and NK cells, which express IL-2Rβγ and are activated and expanded by signalling via this receptor. PIVOT-12 (NCT04410445) is a phase 3 randomised open-label study planning to recruit 950 patients to either adjuvant immunotherapy involving either bempegaldesleukin combined with Nivolumab, or Nivolumab alone, after complete resection of stage III or IV melanoma, using RFS as primary end point. In the first-in-human phase I trial of bempegaldesleukin, it was found to increase activation, proliferation, and PD-1 expression of CD4+ and CD8+ T cells and NK cells [5]. Whether such approaches might be beneficial in late-stage disease is also being pursued. In addition to PIVOT-12, a combination of bempegaldesleukin with Nivolumab is being compared to Nivolumab treatment alone in the advanced disease setting in the PIVOT IO 001 trial (NCT03635983), randomising 764 patients with co-primary end points of overall response rate, progression-free surival (PFS) and OS. Finally, with the aim of enhancing generation of cytotoxic T cells by stimulating intra-tumoural dendritic cell (DC) activation, NCT03435640 is testing the combination of bemppegaldesleukin with NKTR-262, a small molecule TLR7/8 agonist designed to activate antigen-presenting cells (APCs) in the tumour microenvironment, with the bempegaldesleukin/NKTR-262 dual combination being compared to a triplet combination with Nivolumab.

Another IL-2-based approach focuses on RO6874281, an IL2 variant (IL2v) moiety that again abolishes IL2Rα binding but retains IL2Rβγ binding. RO6874281 is bispecific, incorporating a fusion to an antibody binding fibroblast activation protein α (FAPα), a marker selectively expressed on the surface of cancer-associated fibroblasts. This approach should therefore in principle enable retention of RO6874281 in the tumour microenvironment by enabling binding to tumour-associated fibroblasts. In a phase I trial of RO6874281, most adverse events were grade 1/2 [6]. A durable response was observed in a patient with ICI-resistant melanoma and four further melanoma patients showed some tumour shrinkage. As expected from the IL-2 receptor specificity of the agent, there was rapid expansion of peripheral and tumoural CD8 and NK cells, but not T_reg_ cells. A combination strategy is currently being trialled in NCT03875079, involving combining RO6874281 with pembrolizumab, with the percentage of patients undergoing adverse events as the primary end point.

N-803, an IL-15 superagonist, is another engineered cytokine-based therapeutic that is designed to promote both NK and memory/effector T cell stimulation and activation. It consists of a novel IL-15 mutant (N72D), which is generated as a stable heterodimeric complex with the alpha subunit of the IL-15 receptor. The N72D mutation in IL-15 results in a 5-fold increase in biological activity as compared to free IL-15, based on superior binding to the IL-2Rβγ receptor expressed on T and NK cells. This N-803 biotherapeutic is expressed as a structurally modified human IL-15N72D:IL15Rα:IgG1 Fc fusion protein, and exhibits 25-fold higher biological activity and 35-fold longer serum half-life than soluble IL-15. Efforts are now underway to assess the impact of N-803 in the clinic. QUILT-3.055 (NCT03228667) is a multiple cancer indication phase Ib study of N-803 plus various anti-PD-1/PD-L1 mAbs. In five cohorts including melanoma, this doublet (N-803 combined with anti-PD-1/PD-L1) is combined with an NK-based cellular therapy (termed PD-L1 t-haNK), with those patients progressing on the doublet therapy able to transfer to the triplet therapy. PD-L1 t-haNK is a novel NK cell line derived from NK-92 which is engineered to express high-affinity CD16, endoplasmic reticulum-retained IL-2 and a PD-L1-specific chimeric antigen receptor. These retain NK receptors and are highly granzyme B and perforin positive, and appear to lyse myeloid derived suppressor cells, but not other immune cell types, in addition to 20 of 20 cancer cell lines [7]. Cytotoxicity was correlated with PD-L1 expression on target cells, and as expected cytotoxicity could be improved with IFNγ treatment of target cells, which is known to increase PD-L1 expression.

Another NK-based immunotherapeutic of interest is FT500 (Fate Therapeutics), a universal, off-the-shelf NK cell cancer immunotherapy derived from a clonal master induced pluripotent stem cell line. It is hoped that the administration of this allogeneic NK product will overcome ICI resistance associated with the loss of antigen presentation machinery components as a result of acquired mutation. FT500 is now in phase I testing both as monotherapy and in combination with ICI (NCT03841110), for patients with an advanced solid tumour malignancy, including lymphoma. Three cohorts are planned. The first involves FT500 as monotherapy (dose escalation and expansion) in patients who have failed or refused available Food and Drug Administration-approved therapies and are candidates for salvage therapy. A second cohort trials a combination of FT500 and ICI in patients that have progressed on treatment with at least one ICI and who have also failed or refused other available approved therapies and are now candidates for salvage therapy. Finally, there is an FT500 + ICI dose expansion cohort for patients currently receiving Nivolumab, pembrolizumab, or atezolizumab with disease progression on the ICI.

## Targeting immunosuppressive cytokines

In addition to therapeutically harnessing immune potentiating cytokines, inhibition of cytokines associated with immunosuppression is also being explored in clinical trials, with a focus on IL-6 and IL-8 as potential targets. NCT03999749 is a phase II trial of the IL-6 antagonist monoclonal antibody Tocilizumab in combination with Nivolumab and Ipilimumab in ICI-naive unresectable stage III/IV melanoma, which aims to determine the safety, tolerability, and preliminary anti-cancer activity of the combination. Elevated levels of IL-8 are associated with inferior outcomes on ICI therapy, with the most detrimental effect being seen in melanoma patients treated with the combination of Nivolumab with Ipilimumab [8]. Increased tumoural IL-8 levels were associated with lower IFNγ expression, reduced T cell signatures and increased infiltration of myeloperoxidase and/or CD15+ monocytes and neutrophils. To counter this immunosuppressive axis, the anti-IL8 monoclonal antibody BMS-986253 is being pursued in combination with Nivolumab. The dose-escalation component of BMS-986253 is completed and it is understood that planned dose expansion cohorts will be developed based on these results. The trial is currently listed (as of October 2020) as active but not recruiting.

## Personalised vaccines

Given that anti-PD-1 ICI enable microenvironmental antigen-specific T cells, the combination of ICI with neoantigen vaccination strategies is rational and a current avenue of investigation. One such approach, KEYNOTE-942 (NCT03897881), is a randomised phase II trial in resected stage III melanoma comparing the combination of a personalised cancer vaccine termed mRNA-4157 and pembrolizumab, with pembrolizumab alone, incorporating RFS as the primary end point. mRNA-4157 (Moderna) itself is a neoantigen vaccine in which whole-exome DNA and RNA sequencing is used to identify up to 20 human leukocyte antigen-restricted neoantigens. mRNA encoding the neoantigens is then synthesised and encapsulated in lipid nanoparticles before intramuscular delivery, enabling uptake by APC, which then translate the mRNA and present the neo-antigenic peptides on their surface.

An alternative product, RO7198457 (Roche/BioNTech) is a very similar nanoparticulate liposomal mRNA vaccine encoding up to 20 neo-antigens for APC uptake and translation. In this case, the vaccine is delivered intravenously to target DCs in all lymphoid compartments, especially the spleen [9]. Complexing the mRNA in this way protects the mRNA from extracellular ribonucleases, and the approach aims to induce *in situ* DC activation and immune activation in an IFNα mediated manner, via endosomal TLR7 activation of ssRNA in APCs. One trial that is crucial to determining the future of these highly rationally designed neoantigen vaccines is IMCODE001 (NCT03815058), an open-label phase II study in advanced melanoma, which incorporates PFS as the primary end point. It compares a regimen involving RO7198457 combined with Pembrolizumab, with a second arm involving Pembrolizumab alone. Phase I data for RO7198457 were presented at the American Association for Cancer Research 2020. In the monotherapy phase Ia, principally in patients with low/moderate tumour mutational burden, neoantigen-specific T-cell responses were detected via *ex vivo* ELISPOT assays or MHC multimer analyses in 14/16 patients, and T cells against multiple tumour antigens were also detected in post-treatment biopsies [10]. Unfortunately, only 1 of 26 patients responded (a patient with gastric cancer who experienced a complete response). In the phase Ib combination with Atezolizumab (anti-PD-L1), high-level induction of T-cell responses was again observed against the vaccinated neoantigens, but the response rate was only 8% with stable disease in half of the patients [11]. Thus, the randomised melanoma study (NCT03815058) is crucial to assessing whether high-level immune neoantigen reactivity induction translates to clinical benefit in a tumour type associated with anti-PD-1 responsiveness. As an alternative to personalised vaccine approaches based on major histocompatibility complex-restricted neoantigens, it is likely that a BioNTech RNA-LPX vaccine termed Melanoma FixVac, which encodes four non-mutated melanoma tumour-associated antigens (NY-ESO-1, MAGE-A3, Tyrosinase, and TPTE) will also be pursued in the randomised setting following high rates of clinical activity in combination with checkpoint blockade in the recently reported Lipo-MERIT trial [12].

## Adoptive cell therapy with neoantigen-specific T cells

An alternative to neoantigen vaccination is to use neoantigen reactive T cells (NAR-Ts). One of the most compelling approaches in melanoma is the first-in-man THETIS trial (Achilles Therapeutics Inc). It is clear that checkpoint blockade efficacy requires not only sufficient neoantigens but that these neoantigens are clonal [13]. Thus, THETIS exploits tumour as a source for both whole-exome sequencing and production of tumour-infiltrating lymphocytes (TIL). Clonal neoantigens are identified and autologous DCs pulsed with these neoantigens are used to expand TIL recognising these, thereby generating an autologous clonal neoantigen-reactive T cell product, which is delivered to patients following standard Fludarabine/Cyclophosphamide priming and followed by low-dose subcutaneous IL-2. All patients must have received prior ICI. The first patient has been dosed in Newcastle, UK. The CHIRON study is a sister trial in non-small-cell lung cancer.

An alternative to the use of TIL is to engineer autologous T cells with neoantigen-specific T cell receptors (TCRs). NCT03970382 is a study of gene-edited autologous neoantigen-targeted TCR T cells with or without anti-PD-1 ICI or IL-2 in patients with a range of solid tumours including melanoma. NeoTCR-P1 has been developed by PACT Pharma and uses a proprietary technology to capture neoantigen-specific CD8+ T cells from the peripheral blood of the patient after which neoepitope-specific TCRs are cloned and autologous CD8+ and CD4+ T cells engineered to express the relevant TCR. Most NeoTCR-P1 T cells are of T memory stem cell and T central memory phenotypes. Upon antigen recognition, they expand and become highly polyfunctional.

## Targeting the microbiome to enhance checkpoint inhibitor therapy

The influence of the microbiome on ICI outcomes in melanoma and other ICI sensitive cancers has been well documented [14, 15]. Thus, efforts to manipulate the microbiome to enhance outcomes on ICI are rational. One potential agent of interest, SER-401 (Seres Therapeutics), comprises an undisclosed combination of microbes that were selected based on a bacterial signature found in melanoma patients with robust responses to ICI. The MCGRAW study, focussed on unresectable or metastatic melanoma, randomises anti-PD-1 naive patients 2:1 to either SER-401 or matched placebo, each in combination with Nivolumab. The percentage of patients with adverse events is the primary outcome measure and response rate, PFS and OS are secondary end points. An alternative product, MRx0518 (4D pharma plc) is a live preparation of *E. gallinarium* with pre-clinical monotherapy efficacy [16]. Of note, the flagellin of this species appears to impart some of this positive effect on immune reactivity through TLR5 and NF-kB activation [17]. In NCT03637803, MRx0518 is currently being trialled in combination with pembrolizumab in advanced solid cancers including melanoma, in patients who have progressed on anti-PD-1/PD-L1 ICI, with investigation of tolerability and initial signs of clinical benefit.

## Data Availability

There are no novel data generated associated with this review article.

## Abbreviations

APC: Antigen-presenting cells
DC: Dendritic cell
ICI: Immune checkpoint blockade inhibitors
mAbs: monoclonal antibodies
MPR: Major pathologic response
MRD: Minimal residual disease
NK: Natural killer
OS: Overall survival
PFS: Progression-free survival
RFS: Relapse-free survival
TCR: T cell receptors
TIL: Tumour-infiltrating lymphocytes

## Glossary

Nivolumab: anti-PD-1

Pembrolizumab: anti-PD-1

Atezolizumab: anti-PD-L1

Bempegaldesleukin: CD122-preferential IL-2 agonist

RO6874281: IL2v moiety fusion molecule with an antibody binding fibroblast activate protein α

N-803: IL-15 superagonist

PD-L1 t-haNK: NK cell line engineered to express high-affinity CD16, endoplasmic reticulum retained IL-2, and a PD-L1 CAR

FT500: universal, off-the-shelf natural killer (NK) cell cancer immunotherapy derived from induced pluripotent stem cell line

Tocilizumab: anti-IL-6

BMS-986253: anti-IL-8 mAb

mRNA-4157: lipid nanoparticle neoantigen vaccine

RO7198457: nanoparticulate liposomal mRNA vaccine, NeoTCR-P1 - autologous CD8+ and CD4+ T cells engineered to express patient-specific neoepitope-specific TCRs

SER-401: combination of microbes selected based on a bacterial signature found in melanoma patients with robust responses to ICI

MRx0518: live preparation of *E. gallinarium*.

Hazard ratio: the hazard ratio is equivalent to the odds that a patient in the group with the higher hazard reaches the clinical end point first; in a clinical trial assessing relapse-free survival, it represents the odds that a patient in control group will relapse before a patient in the treatment arm.

Open-label trial: a clinical trial in which both researchers and participants are informed about which treatment is being administered.

NY-ESO-1: New York oesophageal squamous cell carcinoma 1

MAGE-A3: melanoma-associated antigen A

TPTE: transmembrane phosphatase with tensin homology

Salvage therapy: a form of therapy provided after the disease does not respond to standard therapy.
